# Artifact propagation in subdural cortical electrostimulation: Characterization and modeling

**DOI:** 10.3389/fnins.2022.1021097

**Published:** 2022-10-12

**Authors:** Jeffrey Lim, Po T. Wang, Susan J. Shaw, Hui Gong, Michelle Armacost, Charles Y. Liu, An H. Do, Payam Heydari, Zoran Nenadic

**Affiliations:** ^1^Department of Biomedical Engineering, University of California, Irvine, Irvine, CA, United States; ^2^Rancho Los Amigos National Rehabilitation Center, Downey, CA, United States; ^3^Department of Neurology, University of California, Irvine, Irvine, CA, United States; ^4^Department of Electrical Engineering and Computer Science, University of California, Irvine, Irvine, CA, United States

**Keywords:** brain-computer interface, bi-directional brain-computer interface, dipole model, electrical stimulation, electrocorticography, stimulation artifacts, subdural stimulation, cortical stimulation

## Abstract

Cortical stimulation *via* electrocorticography (ECoG) may be an effective method for inducing artificial sensation in bi-directional brain-computer interfaces (BD-BCIs). However, strong electrical artifacts caused by electrostimulation may significantly degrade or obscure neural information. A detailed understanding of stimulation artifact propagation through relevant tissues may improve existing artifact suppression techniques or inspire the development of novel artifact mitigation strategies. Our work thus seeks to comprehensively characterize and model the propagation of artifacts in subdural ECoG stimulation. To this end, we collected and analyzed data from eloquent cortex mapping procedures of four subjects with epilepsy who were implanted with subdural ECoG electrodes. From this data, we observed that artifacts exhibited phase-locking and ratcheting characteristics in the time domain across all subjects. In the frequency domain, stimulation caused broadband power increases, as well as power bursts at the fundamental stimulation frequency and its super-harmonics. The spatial distribution of artifacts followed the potential distribution of an electric dipole with a median goodness-of-fit of *R*^2^ = 0.80 across all subjects and stimulation channels. Artifacts as large as ±1,100 μV appeared anywhere from 4.43 to 38.34 mm from the stimulation channel. These temporal, spectral and spatial characteristics can be utilized to improve existing artifact suppression techniques, inspire new strategies for artifact mitigation, and aid in the development of novel cortical stimulation protocols. Taken together, these findings deepen our understanding of cortical electrostimulation and provide critical design specifications for future BD-BCI systems.

## 1. Introduction

Electrocorticography (ECoG) is a viable signal modality for the development of brain-computer interfaces (BCIs). For example, ECoG-based BCIs enabled those with severe motor deficits to communicate (Brunner et al., 2011; Krusienski and Shih, 2011; Vansteensel et al., 2016) or operate an arm prosthesis (Fifer et al., 2013; Wang et al., 2013b). In general, current BCI designs primarily rely on visual feedback to achieve closed-loop operation. However, recent studies (Hiremath et al., 2017; Lee et al., 2018) have demonstrated that electrostimulation *via* subdurally implanted ECoG grids can elicit somatosensory percepts. They suggest that ECoG-based BCIs could carry out both motor and sensory operations, thereby achieving a biomimetic function restoration. Preliminary results also indicate that such bi-directional operation can improve BCI performance by easing the learning process and making this technology more intuitive (Flesher et al., 2017). Finally, subdural ECoG-based interfaces also have the potential to be designed as fully implantable systems (Geller, 2018), which could significantly improve the viability of ECoG-based BCIs.

Bi-directional (BD)-BCI operation requires simultaneous stimulation and recording, which poses a significant challenge as strong electrical artifacts will inevitably propagate from the stimulation site to the recording site. These artifacts can obscure physiologically relevant signals or even saturate ultra-low-power (ULP) analog front-ends, which are necessary for the implementation of fully-implantable BCIs (Stanslaski et al., 2009; Rouse et al., 2011). Therefore, efficient artifact suppression strategies must be developed and employed to mitigate this problem.

Microelectrode-based BD-BCIs (O'Doherty et al., 2011; Flesher et al., 2017) address this problem by temporally interleaving stimulation and recording. However, this approach is suboptimal since it records data intermittently, thereby degrading the performance of BCI decoding algorithms. Additionally, such a BD-BCI system can only provide intermittent feedback. Motivated by these shortcomings, several groups developed alternative approaches. For example, Stanslaski et al. proposed to orient the ECoG stimulation electrodes in a way that minimizes the artifact presence at the recording site (Stanslaski et al., 2009). Subsequently, they applied frequency-domain filtering to remove the residual artifacts (Stanslaski et al., 2009; Rouse et al., 2011). An adaptive filtering approach proposed by Mendrela et al. (2016) achieves artifact suppression by estimating the artifact contributions and then subtracting those contributions from the signal at the front-end. Barborica et. al have utilized an alternating polarity stimulation method that minimizes the power at the stimulation frequency and its super-harmonics (Barborica et al., 2022). Recording techniques like bipolar montages can also be used to minimize common mode artifacts between electrodes (Perrone-Bertolotti et al., 2020), though this method is only applicable to artifacts coming from distant sources. Our group has also recently developed an artifact suppression method based on an auxiliary stimulator that steers artifacts away from the recording site (Lim et al., 2019; Pu et al., 2020). The advantage of this approach is that we suppress artifacts before they reach the recording site. While fundamentally different, these artifact mitigation strategies critically depend on factors such as the relative orientation and the distance between the stimulation and recording channels, the stimulation parameters, and the electrical properties of the relevant tissues and the electrode-tissue interface. A better understanding of these factors can improve existing artifact suppression techniques, inspire new strategies for artifact mitigation, and aid the development of novel cortical stimulation protocols.

Despite the need, there have been very few attempts to characterize artifacts resulting from ECoG stimulation. This is surprising given the prevalence of clinical ECoG mapping in epilepsy surgical evaluation (Phase II epilepsy monitoring). Motivated by this knowledge gap, our preliminary work (Lim et al., 2018) characterized ECoG stimulation artifacts in a single human subject. We also hypothesized that a simple dipole model might explain the spatial distribution of these artifacts, as previous works suggest that conduction of neural signals through neural tissue follows dipole volume conduction (Wood, 1982; Scherg, 1990; Boon et al., 1997; Sutherling et al., 2001; Nunez and Srinivasan, 2006). In this article, we present the extension of these preliminary findings to four subjects, by performing a comprehensive analysis of ECoG stimulation artifacts in the temporal, frequency, and spatial domains. Additionally, we conducted a modeling study and found an electric dipole to be an accurate model of the stimulation artifact propagation across multiple human subjects with varying implantation sites and ECoG electrode types. Collectively, these findings deepen our understanding of cortical electrostimulation and provide critical design specifications for future BD-BCI systems.

## 2. Methods

### 2.1. Subject information and stimulation procedure

The Institutional Review Boards of the Rancho Los Amigos National Rehabilitation Center and the University of California, Irvine approved this study. We conducted all research procedures according to the Declaration of Helsinki. Four patients undergoing Phase II epilepsy monitoring gave written informed consent to participate in the study. Subject 1 and 3 were implanted with platinum ECoG grids (Ad-Tech, Oak Creek, WI). Subjects 2 and 4 had platinum-iridium ECoG grids (Integra Life-Sciences, Plainsboro, NJ) implanted. The placement and number of ECoG grids/strips, as well as the choice of stimulation electrodes were solely guided by clinical needs. We only analyzed the stimulation epochs from grids that had all electrodes stimulated (representative grids). Other implanted grid/strips were excluded because they had either incomplete or no stimulation coverage.

We recorded clinical ECoG data at the bedside during eloquent cortex mapping procedures. These procedures are a standard part of Phase II epilepsy monitoring and entail electrostimulation of cortical tissue across sequential channels of the ECoG grids. A bipolar stimulation channel consisted of a pair of adjacent electrodes connected to a Natus^®^Nicolet^TM^ Cortical Stimulator (Natus Medical Incorporated, Pleasanton, CA). For each stimulation channel, the stimulator delivered a biphasic square pulse train with equal-length anodic/cathodic pulse width ranging from 200 to 250 μs over a short stimulation epoch. The duration of stimulation epochs varied from 2 to 5 s across subjects. The stimulation amplitude also varied from 2 to 12 mA, typically in 2 mA increments. Note that the choice of stimulation channels and parameters was solely guided by clinical needs. See Table 1 for a comprehensive list of stimulation parameters and representative grid information for each subject. We acquired ECoG data at 512 Hz using a Natus^®^Quantum^TM^ amplifier (Natus Medical Incorporated, Pleasanton, CA) during the entire mapping procedure and annotated stimulation channels and epochs. This amplifier had a ± 3 dB linear range 0.01–219 Hz for 512 Hz sampling rate.

**Table 1:** Stimulation parameters and representative grid information.

**Stim./grid**	**Subject 1**	**Subject 2**	**Subject 3**	**Subject 4**
**parameters**	
Stimulation frequency (Hz)	50	50	50	50
Pulse width (μs)	200	250	200	250
Epoch duration (s)	3	5	2	2
Amplitude (mA)	2-8	2-10	3-12	2-12
Grid vendor	Integra	Ad-Tech	Integra	Ad-Tech
Grid type	Standard	Standard	High Density	High Density
Electrode spacing (mm)	10	10	3	4
Electrode diameter (mm)	4.75	4.00	2.00	2.00
Exposure diameter (mm)	1.5	2.3	1.0	1.0
Reference/ground	LPG1/2	LTG19/20	Off-grid	Off-grid

### 2.2. MR-CT image segmentation and co-registration

To aid in the artifact propagation characterization, we first determined the coordinates of the ECoG electrodes in reference to the brain. Specifically, we used pre-implantation MRI (post-explantation MRI for Subject 4) and post-implantation CT images to co-register the ECoG electrodes with brain-segmented MR images. For this purpose, we used the Elastix toolbox (Klein et al., 2010; Shamonin et al., 2014), which performs non-rigid co-registration of MRI and CT images. We used default parameters and a normalized mutual information similarity metric. We fixed the CT image to preserve the electrode coordinates, while moving the MR image until it was transformed to the CT coordinate space. We then segmented the transformed MRI using the Mango (Lancaster et al., 2010, 2011, 2012) segmentation plugin to prepare for co-registration with the electrode coordinates segmented from the CT images.

The segmentation of the electrode coordinates from subject post-implantation CT data followed the procedure described in Wang et al. (2013a). First, to identify the electrode locations, we thresholded the CT intensity data. For each electrode location, this procedure generated an intensity point cluster. Subsequently, we ran a clustering algorithm utilizing DBSCAN (Ester et al., 1996) that returned the center-point for each electrode in the array. These CT-space electrode coordinates were then overlaid onto the transformed MRI-segmented brain to complete the co-registration process. Finally, we scaled the coordinates by the CT image voxel dimensions (mm) to convert from voxels to physical space.

### 2.3. Time domain analysis

We collected ECoG data at the bedside during cortical electrostimulation procedures. Each time the subject received stimulation, we timestamped the corresponding stimulation epoch so that these data could be subsequently identified and segmented out for further analysis. For each subject, this procedure generated several hours of data. We used MATLAB (MathWorks, Natick, MA) for data processing and analysis. The signals were first visually inspected to assess the quality of the baseline ECoG data and confirm the presence of stimulation artifacts. Subsequently, for each channel, we removed low-frequency drifts by high-pass filtering at ≥1.5 Hz (zero-phase, first-order, Butterworth filter). Note that this filter had negligible effects on the rest of the signal (see **Figure 2**). We then segmented the individual stimulation epochs from the rest of the data using the documented timestamps. The electrodes comprising the stimulation channel were excluded from the analysis due to amplifier saturation. Any electrodes with signals exceeding the clinical amplifier's saturation limit (±8.7 mV) were also excluded. For each remaining electrode, we identified the responses to individual biphasic pulses. Depending on the duration of the stimulation epoch (see Table 1), this procedure resulted in 100–250 pulse responses per stimulation epoch. Within each stimulation epoch and for each electrode, we then quantified the artifact amplitude by finding the extreme value of each pulse response. This procedure was aided by utilizing the known pulse train frequency (50 Hz) and MATLAB's extrema detection algorithm.

### 2.4. Frequency domain analysis

To take advantage of the periodic nature of the stimulation signals and corresponding ECoG responses, we also analyzed data in the frequency domain. To this end, we divided each stimulation epoch into five equal, non-overlapping segments. We then performed the fast Fourier Transform (FFT) on each data segment and calculated their power spectral densities (PSDs). We repeated the same procedure for baseline epochs, defined as duration-matched periods immediately preceding the corresponding stimulation epochs. To quantify the effect of the stimulation across frequency, *f*, we calculated the interference index, *I*(*f*), as:


(1) $$\begin{array}{l} I\left(f\right)=\frac{1}{2}\log\frac{\sigma_{\text{t}}^{2}\left(f\right)}{\sigma_{\text{on}}\left(f\right)\sigma_{\text{off}}\left(f\right)} \end{array}$$

where σ_on_(*f*) and σ_off_(*f*) are the frequency-dependent standard deviations of the stimulation and baseline PSDs, respectively, and $\sigma_{\text{t}}^{2}\left(f\right)$ is the total variance calculated as Fukunaga (2013):


(2) $$\begin{array}{l} \sigma_{\text{t}}^{2}(f)=\frac{\sigma_{\text{on}}^{2}(f)+\sigma_{\text{off}}^{2}(f)}{2}+\frac{{\left[\mu_{\text{on}}(f)-\mu_{\text{t}}(f)\right]}^{2}}{2}\\ \;+\frac{{\left[\mu_{\text{off}}(f)-\mu_{\text{t}}(f)\right]}^{2}}{2} \end{array}$$

In the above equation, μ_on_(*f*) and μ_off_(*f*) are the frequency-dependent means of the stimulation and baseline PSDs, respectively, and μ_t_(*f*) is the total mean calculated as: $\mu_{\text{t}}\left(f\right)=\frac{1}{2}\left[\mu_{\text{on}}\left(f\right)+\mu_{\text{off}}\left(f\right)\right]$. Note that Equation (1) is a variant of the deflection coefficient (Kay, 1989) that can account for overlapping means and unequal variances between the stimulation-on and stimulation-off PSDs (Nenadic, 2007). We also compared the power distribution in the stimulation-on and stimulation-off conditions across frequencies and tested the statistical significance of these differences by performing the Kolmogorov-Smirnov (KS) test.

### 2.5. Spatial domain analysis

Based on the artifact amplitudes calculated in the time domain analysis, we characterized each stimulation epoch by calculating its median artifact amplitude. We repeated this procedure for each electrode in the grid, and then interpolated and color-coded these median values to generate artifact spatial maps. From these maps, we defined a saturation region as the cortical area within which artifacts were large enough to saturate a hypothetical ULP amplifier. To this end, we first derived a saturation limit from the specifications of an implantable bi-directional BCI prototype (Rouse et al., 2011). Specifically, a supply voltage of 2.2 V and a gain of 66 dB (2000×) yielded a saturation limit of ±1,100 μV. We then marked this saturation limit as a contour on the artifact spatial maps and defined the contour interior as the saturation region. Finally, we quantified the extent of the saturation region by calculating the worst-case distance (WCD), defined as the maximum distance between the mid-point of the stimulation channel and the saturation contour.

### 2.6. Dipole model analysis

Our preliminary work suggests that the spatial distribution of artifacts follows the voltage distribution of an electric dipole (Lim et al., 2018). To test this hypothesis, we estimated a dipole model from ECoG measurements and assessed its accuracy using an R-squared value. We then used the model to predict spatial distributions of artifacts and compared them to distributions generated from experimental data.

The spatiotemporal distribution of potentials due to a dipole in a homogeneous, isotropic, purely resistive medium is given by Logothetis et al. (2007):


(3) $$\begin{array}{l} \phi \left(x,y,z,t\right)=\frac{I\left(t\right)}{4\pi \sigma }\left(\frac{1}{\left||r\left(x,y,z\right)-r_{+}|\right|}-\frac{1}{\left||r\left(x,y,z\right)-r_{-}|\right|}\right) \end{array}$$

where ϕ(*x, y, z, t*) is the potential field at a point (*x, y, z*) and time *t*, generated by a pair of positive and negative, time-dependent, point current sources with the position vectors $r_{+}\in {\mathbb{R}}^{3}$ and $r_{-}\in {\mathbb{R}}^{3}$, respectively. The vector *r*(*x, y, z*) ∈ ℝ^3^ defines the position of the point (*x, y, z*). Note that we defined these position vectors, *r*_+_, *r*_−_, and *r*, with respect to an arbitrarily chosen origin and that the choice of origin is irrelevant due to the homogeneity and isotropy assumptions. For the justification of cortical tissue behaving as a homogeneous, isotropic, purely resistive medium, we refer to Ranck (1963), Nicholson and Freeman (1975), Okada et al. (1994), and Logothetis et al. (2007). Finally, σ is the conductivity of the medium and *I*(*t*) is current of the source/sink.

Taking the above considerations into account, the dipole equation can be reformulated as:


(4) $$\begin{array}{l} V_{e}=kI\left(\frac{1}{\left||r_{e}-r_{+}|\right|}-\frac{1}{\left||r_{e}-r_{-}|\right|}\right)+n,\;e=1,2,\cdots \;,N_{e} \end{array}$$

where *V*_*e*_ is the artifact amplitude measured at electrode *e*, *N*_*e*_ is the number recording electrodes, and *I* is the amplitude of the stimulation current. The position vectors *r*_*e*_, *r*_+_, and *r*_−_ define the positions of the electrode *e*, the current source, and the current sink, respectively. We calculated these position vectors from the CT images with respect to an arbitrary origin. The slope parameter *k* accounts for the geometry of the current transmission path, the electrode size and material, and the impedance of the electrode-tissue interface. Finally, the intercept parameter *n* accounts for the placement of the reference electrode, background neural activity, and environmental noise. Note that the choice of the reference electrode was determined clinically (see Table 1). Generally, its position was not on a zero-potential line, defined theoretically as a line equidistant to the dipole source and sink. This may have contributed to a non-zero reference voltage that needs to be accounted for by *n*.

For a given dipole location, we measured the artifact amplitudes, *V*_*e*_, at multiple stimulation currents, *I*, and estimated the parameters *k* and *n* in Equation (4) using a linear least-squares approach. We also quantified the goodness-of-fit using the R-squared value. Once the parameters, $\hat{k}$ and $\hat{n}$, are estimated, we predicted the spatial distribution of artifacts, ${\hat{V}}_{e}$, using the model Equation (4). Finally, we interpolated the predicted artifact values and then mapped them onto the MR-CT co-registered images for visualization purposes.

## 3. Results

### 3.1. Co-registration

**Figure 1:**
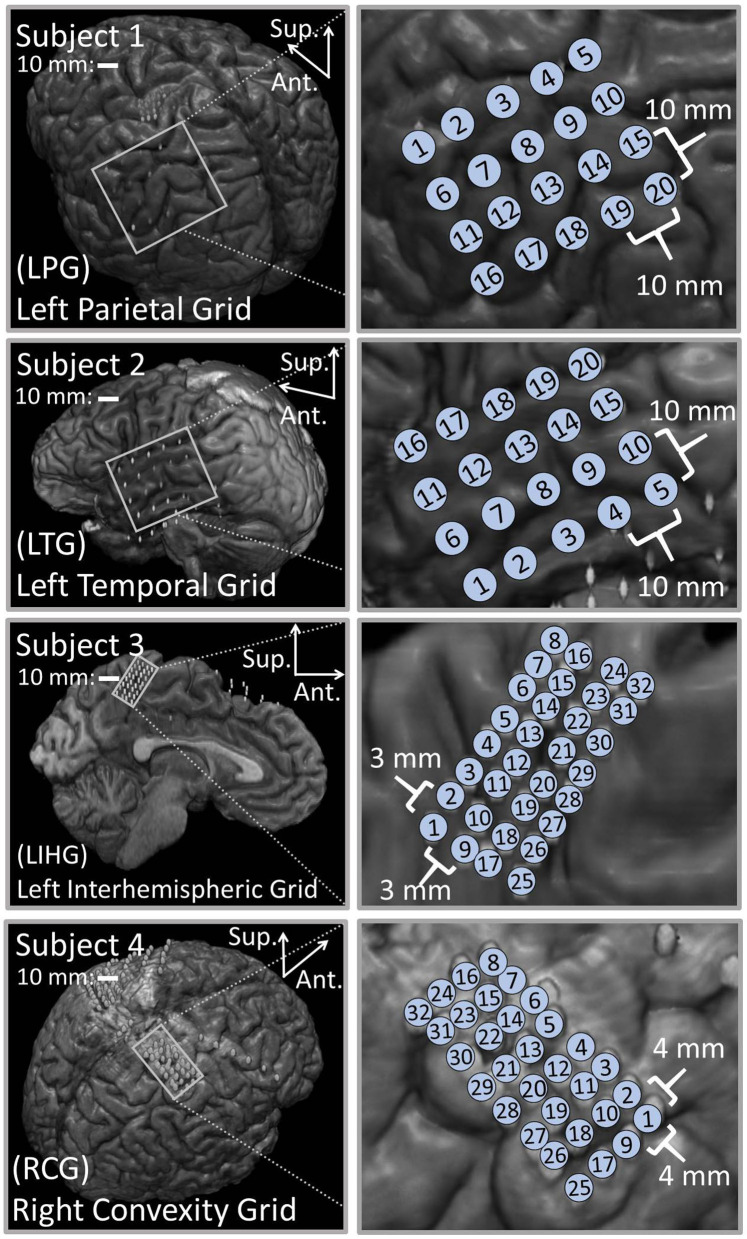
Co-registration of electrodes segmented from CT images and brain segmented from MRI. **(Left)** Co-registration image for each subject, with the representative grid outlined in white. Note that the points representing electrodes are not to scale. Also note that the co-registration image for Subject 3 shows a left hemisphere (sagittal plane) with electrodes in the inter-hemispheric fissure. **(Right)** Insets of representative grids with electrode numbers encircled. The label size is not related to the electrode size.

Figure 1 shows the results of the co-registration procedure for all four subjects. Subsequently, we used these co-registration images for visualization purposes and spatial domain analysis. While we analyzed a single representative grid for each subject (see Table 1), we still obtained abundant data, with 6–21 dipoles per grid and 2–7 stimulation amplitudes per dipole. The analyzed grid for Subject 1 was a 4×5 standard grid implanted over the left, posterior parietal area. Subject 2 had a 4×5 standard grid implanted over the left temporal lobe. Subject 3 had a 4×8 high-density grid in the interhemispheric space over the left motor leg area. Finally, Subject 4 had a 4×8 high-density grid placed over the right motor arm area.

### 3.2. Time domain analysis

Visual inspection of the ECoG data at various time scales revealed salient features of stimulation artifacts. Looking on a minutes time scale, we easily identified the individual stimulation epochs, since stimulation created significant artifacts in the ECoG data across multiple electrodes.

**Figure 2:**
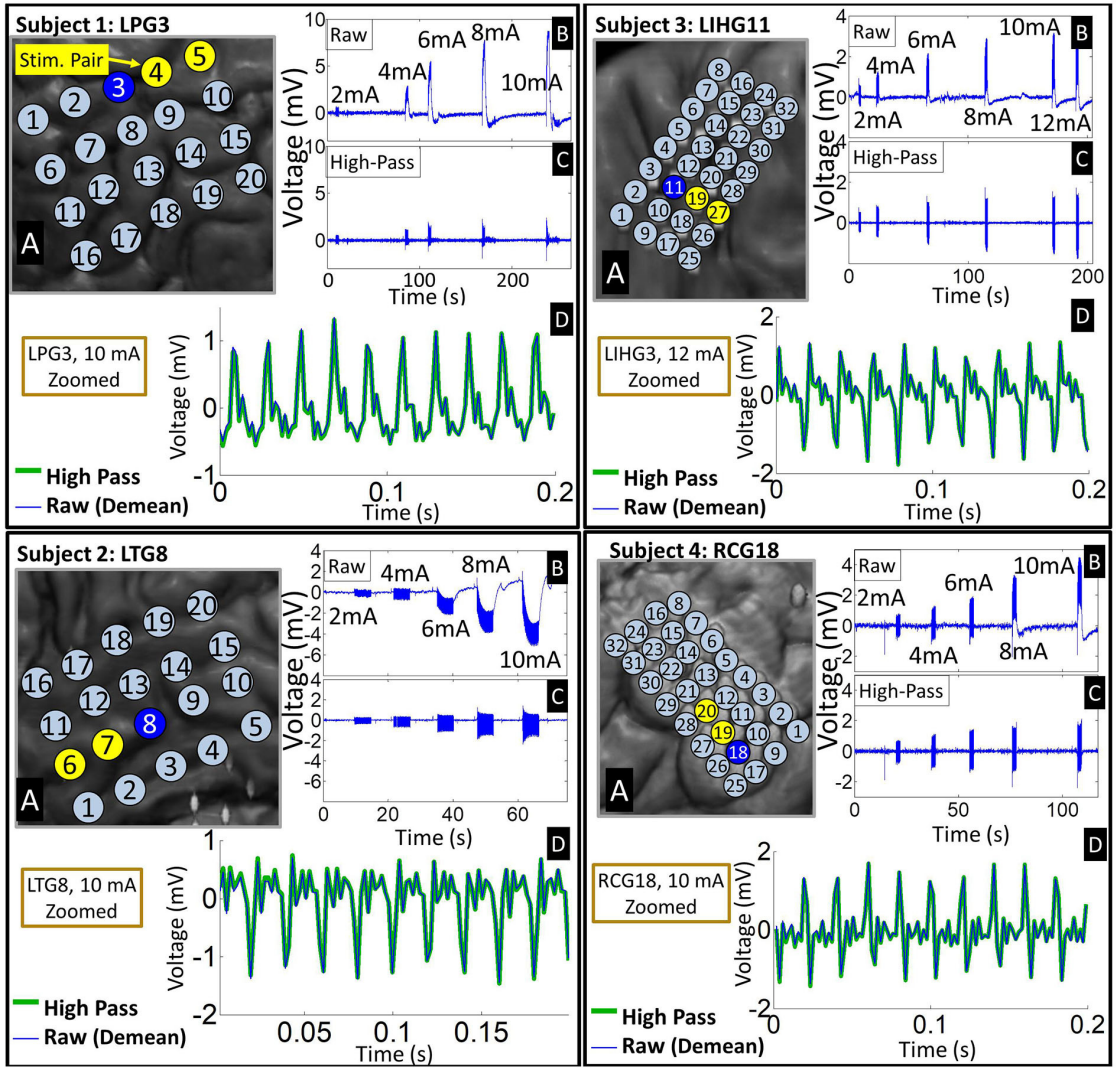
Representative examples of time-domain signal features across four subjects. **(A)** Insets of co-registered images showing the representative grid with the stimulation channel marked in yellow. **(B)** Time-domain signals from an electrode (marked by blue on the grid) adjacent to the stimulation channel exhibiting ratcheting effects. The raw waveforms show 5–6 stimulation epochs at different stimulation amplitudes. Note that ratcheting severity increases with stimulation amplitude. **(C)** The ratcheting is removed by high-pass filtering at 1.5 Hz. **(D)** A zoomed plot of the strongest artifacts before and after filtering shows that high-passing has negligible effect on the individual pulse responses. Raw signal is de-meaned so that it can be overlaid with high-passed signal.

On a seconds time scale, we observed large voltage deviations in the millivolts range during the stimulation epochs, particularly on electrodes close to the stimulating channel. These deviations are significantly larger than typical ECoG signals, which have an amplitude of 10's of microvolts (Schalk and Leuthardt, 2011; Wang et al., 2017). Despite the supposed charge balance of the biphasic square waveform, these electrodes accumulated a significant DC drift over the duration of the stimulation epoch (see Figure 2). This so-called “ratcheting effect” (Merrill et al., 2005) was prominent in the raw ECoG data across all four subjects. The voltage drifts caused by the ratcheting effect generally exceeded the amplitude of the pulse responses by several fold, and occasionally drove electrodes adjacent to the stimulation channel to the saturation voltage of the recording system amplifier (±8.7 mV). High-pass filtering at 1.5 Hz removed the ratcheting effect on non-saturated electrodes. Note that the polarity of the DC drift on ratcheting electrodes depended on their position with respect to the current source/sink at the onset of the stimulation.

**Figure 3:**
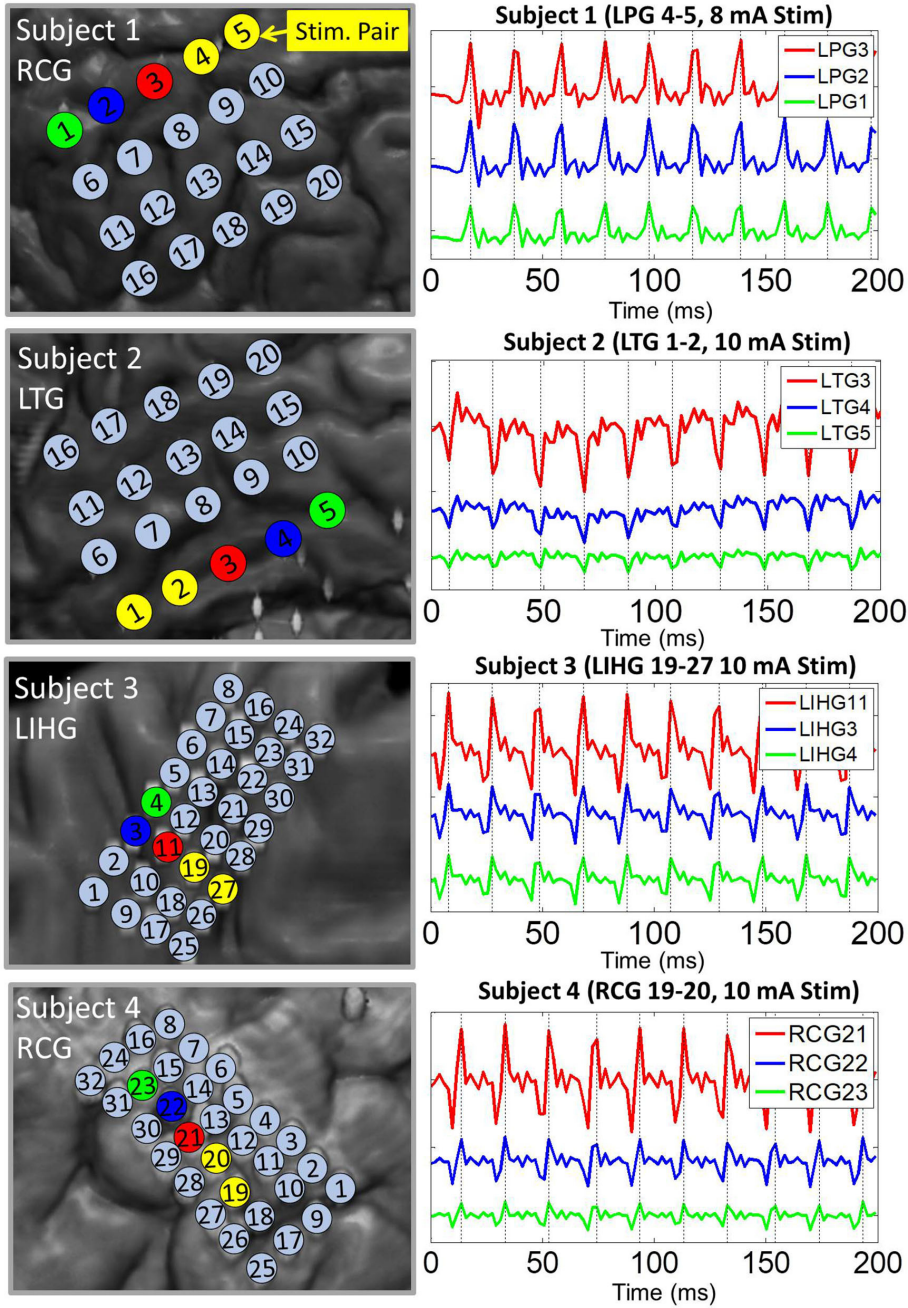
Representative examples of pulse response phase-locking in all 4 subjects. **(Left)** Insets of co-registration images, with color-coded electrodes lying in the direction of strongest artifact (co-linear with the dipole moment). **(Right)** ECoG data high-passed at 1.5 Hz with colors matched to the corresponding electrode. Artifact peaks/troughs, marked by vertical lines, on these electrodes are within 2 ms (1 sample) of each other. Pulse responses occur every 20 ms (50 Hz pulse train frequency). The artifact amplitude decreases with distance from the stimulation channel.

Pulse responses across electrodes exhibited phase-locking on a milliseconds time scale, especially at higher stimulation amplitudes. More specifically, voltage peaks/troughs on artifact-affected electrodes occurred within 2 ms (1 sample) of each other (see Figure 3). This behavior was consistent across stimulation channels and grids. For all four subjects, the frequency of the pulse responses matched the frequency of the stimulation pulse train (50 Hz). These observations are consistent with the assumptions of the dipole model given by Equation (4).

### 3.3. Frequency domain analysis

**Figure 4:**
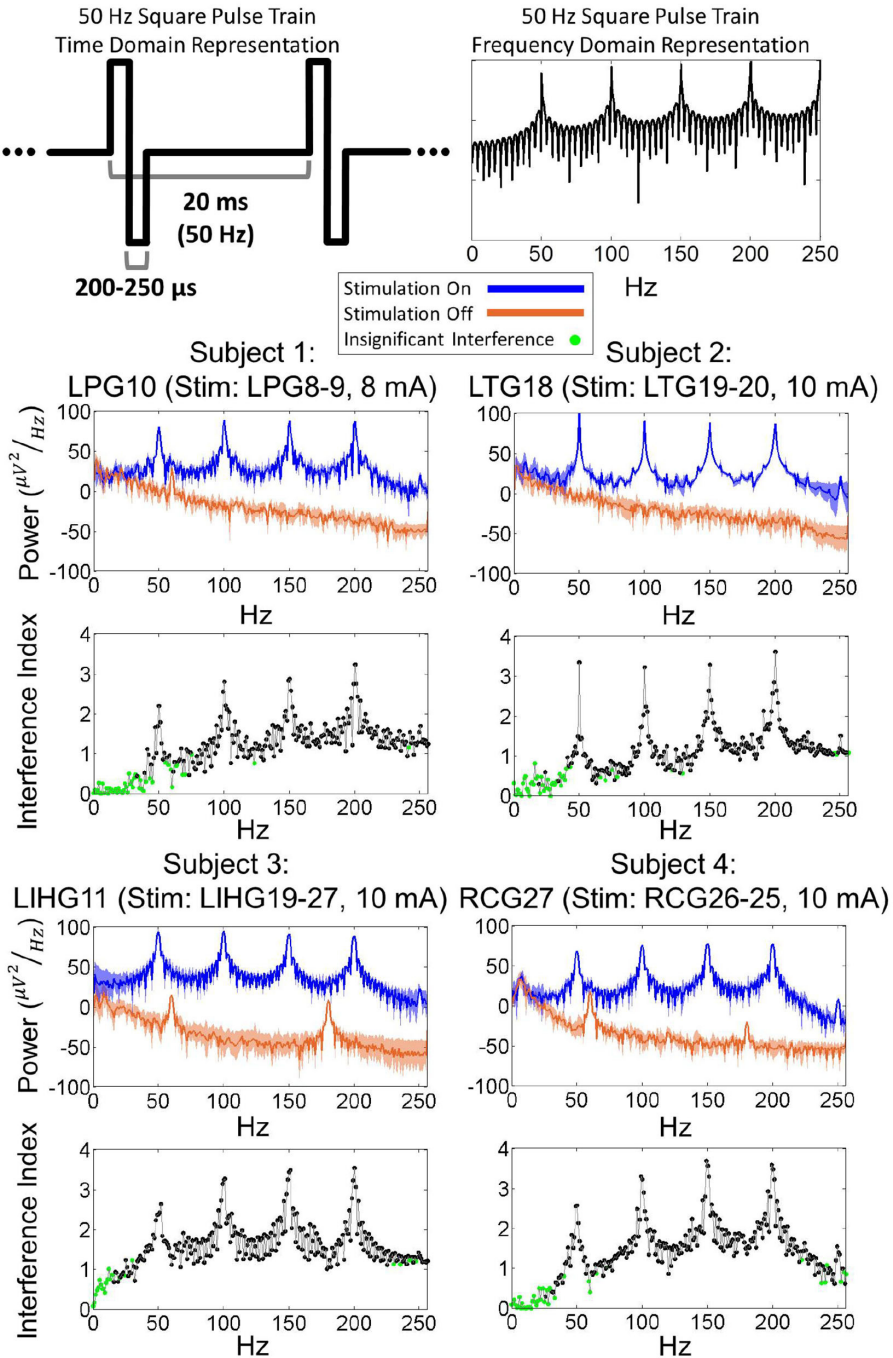
**(Top)** Time domain and frequency domain representations of a 50 Hz square pulse train. **(Bottom)** Power spectra of ECoG signals during 50 Hz biphasic square pulse stimulation for worst-case electrodes from each subject. The power distribution of ECoG signals, with peaks present at 50, 100, 150, 200 Hz, resembles the power distribution of a 50 Hz square pulse train. Interference index and Kolmogorov-Smirnoff testing (*p* = 0.01) show that a majority of significantly impacted frequencies are above the 50 Hz stimulation pulse frequency.

Figure 4 shows examples of the power spectral density (PSD) for each subject's worst-case electrode, which are defined as electrodes experiencing the strongest artifact for a given stimulation channel. Other electrodes exhibited similar PSDs as the worst-case electrode, albeit with lower overall power. By comparing the stimulation-on and stimulation-off PSDs, it is evident that the stimulation induced a significant broadband power increase. Additionally, there were prominent power peaks at the fundamental frequency of the stimulation pulse train (50 Hz) as well as its super-harmonics (100, 150, 200, 250 Hz). Frequencies below 50 Hz exhibited lower interference index values for all 4 subjects. These results were corroborated by the KS test (*p* = 0.01), which showed that the stimulation-on and stimulation-off PSDs were not significantly different for a majority of frequencies below 50 Hz. Other electrodes in the grid exhibited similar PSDs, albeit with reduced overall power.

### 3.4. Spatial domain analysis and dipole model estimation

**Figure 5:**
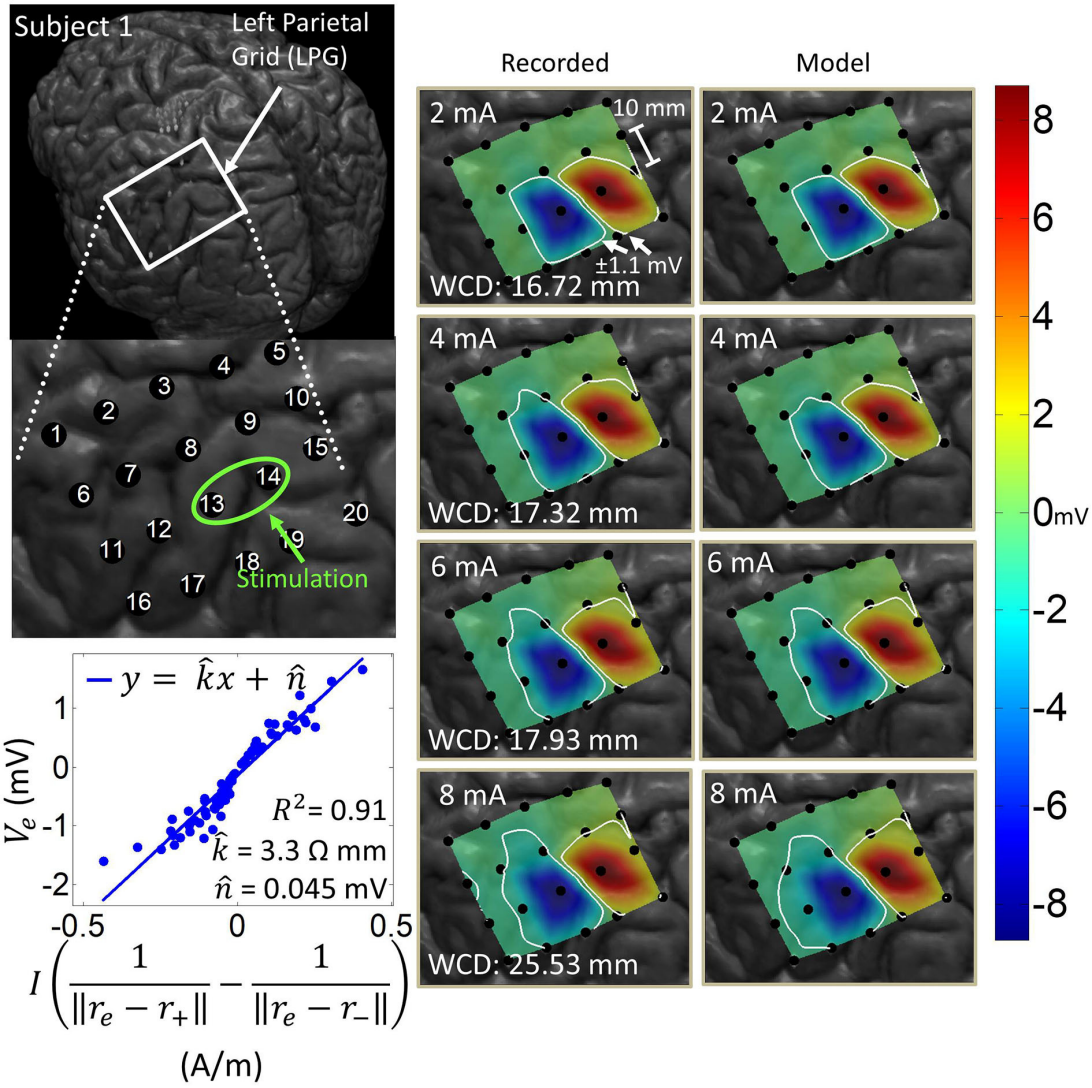
Artifact spatial maps based on recorded ECoG data and model predictions for Subject 1. **(Top left)** Co-registration image showing the representative grid over the left parietal lobe. **(Bottom left)** Regression results aggregating data from stimulation channel LPG13-14 for 2–8 mA stimulation amplitudes. Artifact spatial maps were generated using values from the recorded data **(middle)** and the dipole model **(right)**. The worst-case distance (WCD) is the distance from the center of the stimulating channel to the farthest point on the ±1.1 mV contour. Note that the electrodes comprising the stimulation channel (LPG13-14) were saturated on the hospital ECoG recording system and recorded no data. As such these were excluded from the dipole regression and their values were set to ±8.711 mV (the saturation limit of the hospital ECoG recording system) on the artifact spatial maps.

**Figure 6:**
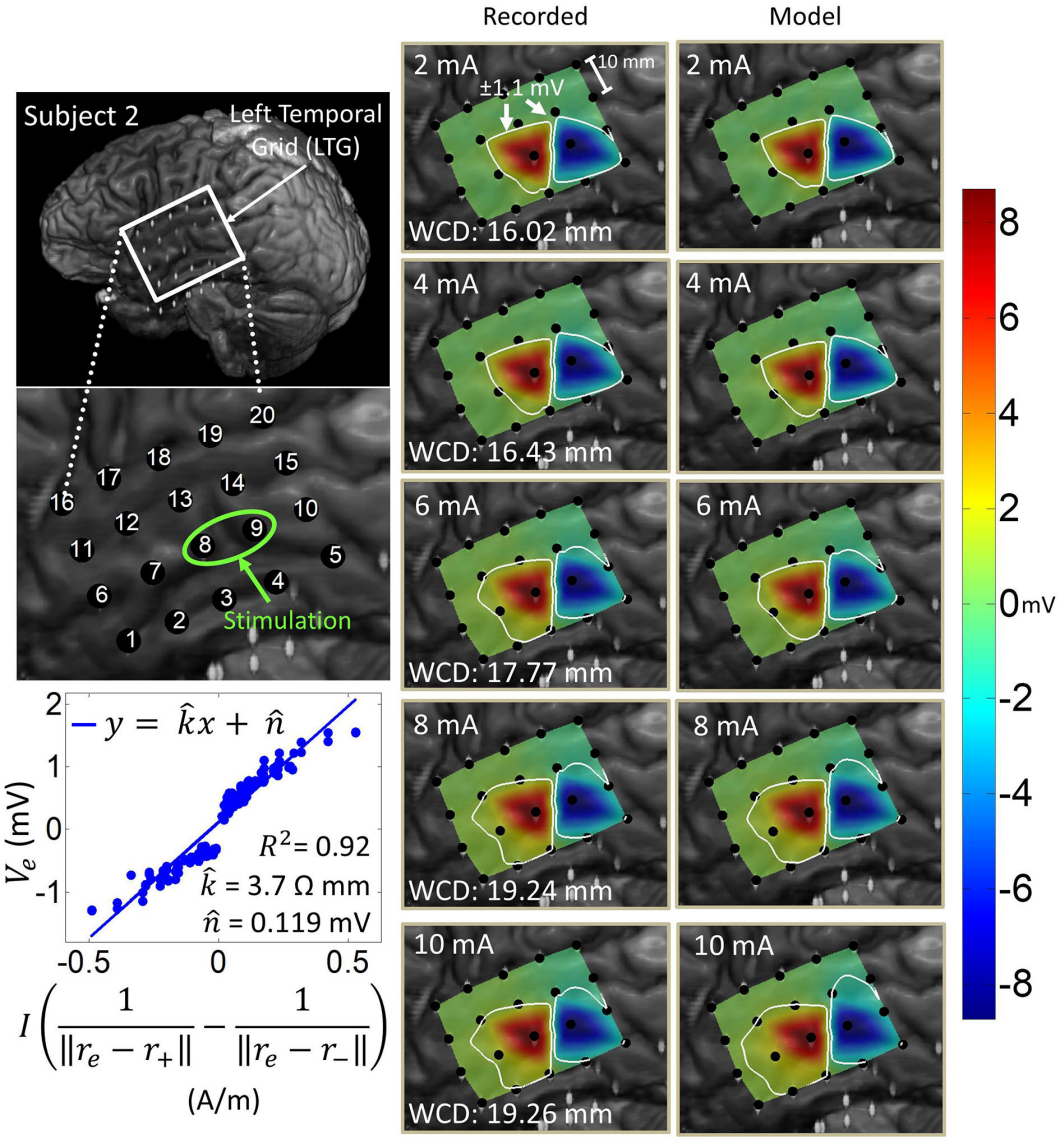
Artifact spatial maps for recorded ECoG data and model predictions for subject 2. **(Top left)** Co-registration image showing the representative grid over the left temporal lobe. **(Bottom left)** Regression uses ECoG data from 2 to 10 mA stimulation amplitudes on the stimulation channel LTG8-9. Artifact spatial maps were generated using values from the recorded data **(middle)** the dipole model **(right)**. Electrodes LTG19 and LTG20 contained no recorded ECoG data, so they are excluded from the analysis (values on the recorded data map are imputed from electrode LTG18).

**Figure 7:**
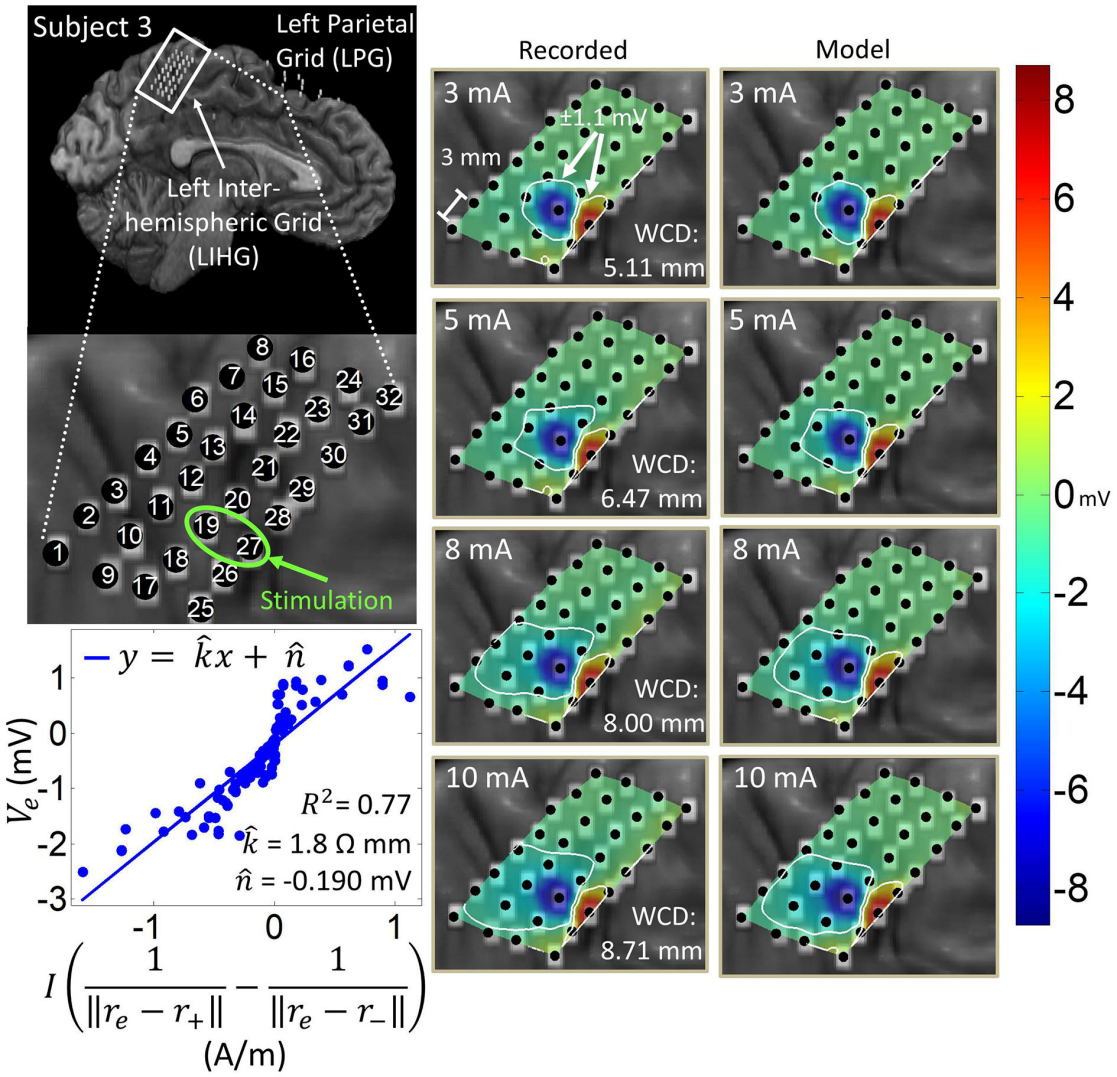
Artifact spatial maps for recorded ECoG data and model predictions for subject 3. **(Top left)** Co-registration image showing the representative grid located over the leg area on the left side of the interhemispheric fissure. **(Bottom left)** Regression results for the stimulation channel LIHG19-27 aggregating data from current amplitudes 3–10 mA. Artifact spatial maps were generated using values from the recorded data **(middle)** the dipole model **(right)**.

**Figure 8:**
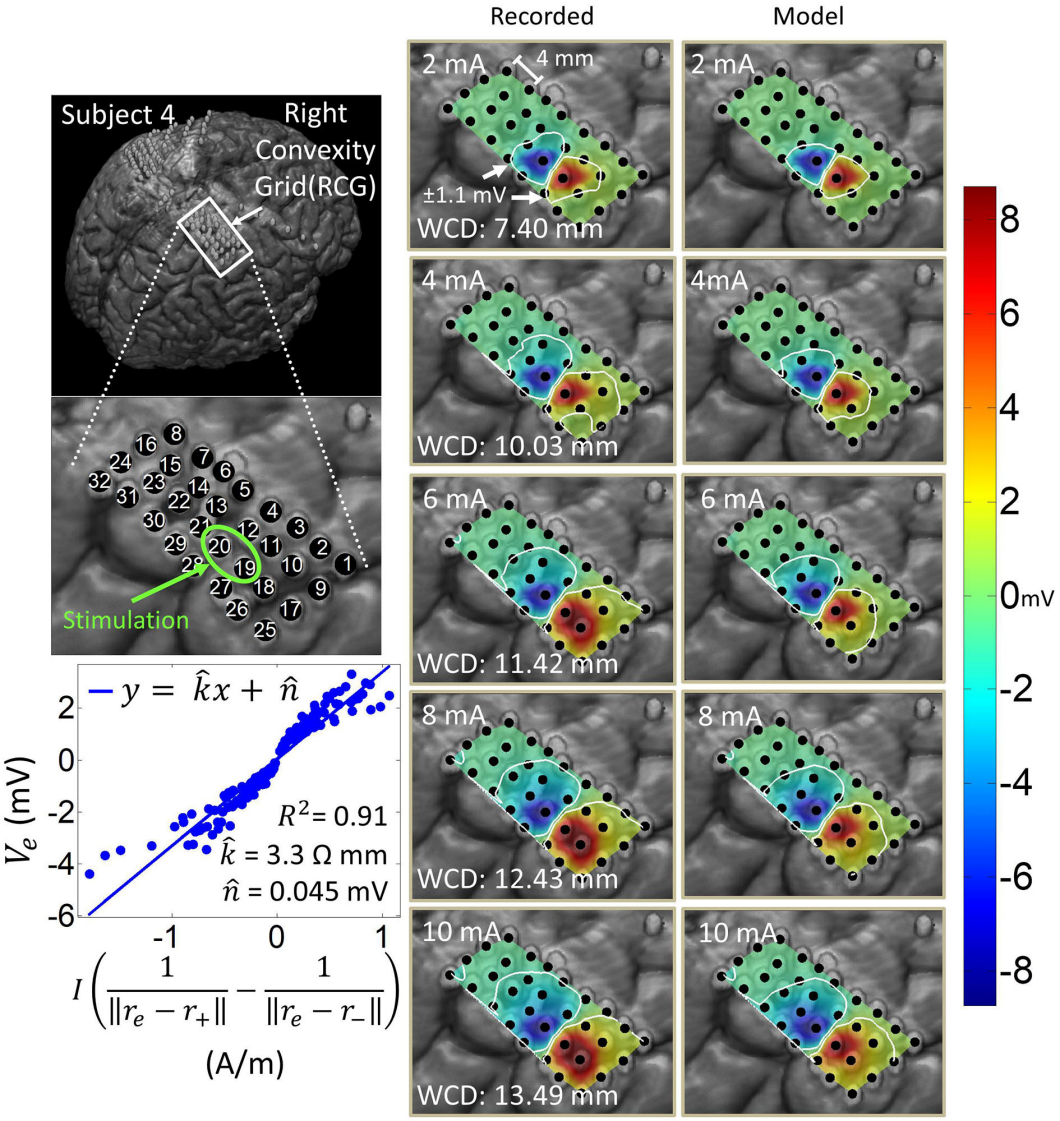
Artifact spatial maps for recorded ECoG data and predicted data for subject 4. **(Top left)** Co-registration image showing the representative grid located over the right sensorimotor area. **(Bottom left)** Regression results for the stimulation channel RCG19-20 aggregating data from current amplitudes 2–10 mA. Artifact spatial maps were generated using values from the recorded data **(middle)** the dipole model **(right)**. Electrode RCG18 saturates at 6 mA and above, at which point it is excluded from the analysis as it no longer contains any ECoG data.

Artifact spatial maps exhibited dipole-like voltage distributions, representative examples of which are shown in Figures 5–8 for each subject. The amplitude of a pulse response recorded by an electrode depended on the position of that electrode with respect to the stimulation channel. Generally, the artifact amplitude scaled inversely with the distance of the electrode to the stimulating channel, which is consistent with Equation (4). Furthermore, electrodes lying co-linearly with the stimulation dipole moment experienced stronger artifacts compared to those lying orthogonally. Also, artifact amplitudes increased monotonically with stimulation amplitude, which resulted in the expansion of the saturation region. Correspondingly, the worst-case distance also monotonically increased with stimulation amplitude for these examples. Some exceptions to these behaviors occurred at higher stimulation amplitudes, where the increased current caused a departure from the dipole voltage distribution.

The ranges of WCDs for each subject over all stimulation channels and amplitudes are reported in Table 2. For a comprehensive list of all WCDs across all subjects, stimulation channels, and stimulation amplitudes, the reader is referred to the Supplementary material. We found the saturation regions to be localized to the vicinity of the stimulation channel, with WCDs ranging from 4.43 to 38.34 mm. Generally, larger WCDs corresponded to larger stimulation amplitudes. The resolution of the WCD is limited by the relatively coarse spatial resolution of ECoG electrodes within a grid. Other factors, such as grid placement and electrode material properties may have also affected the WCD values. Another contributing factor is the location of the stimulation channel within the grid, which may limit the ability for the dipole field to be captured entirely, e.g, stimulation channels placed on the corner or the edge of the grid.

**Table 2:** WCD ranges for each subject.

**Subject no**.	**Min. WCD**	**Max. WCD**	**Stim. range**
	**(mm)**	**(mm)**	**(mA)**
Subject 1	12.06	38.34	2–10
Subject 2	12.43	36.45	2–12
Subject 3	4.43	9.12	3–12
Subject 4	5.40	25.89	2–12

Table 3 shows the median and median absolute deviation (MAD) for parameters of the linear regression model and goodness-of-fit values across different stimulation channels for each subject. We chose median-based statistics to counter the effect of a few outliers, primarily contributed by stimulation channels located at the corners of ECoG grids. Despite the differences in implantation site and grid size/type, the median values of $\hat{k}$ remained relatively consistent across subjects and ranged from 2.1 to 3.9 Ωmm. Similarly, the median values for $\hat{n}$ were consistent across subjects, and ranged from -78 μV to 70 μV. These values are within the same order of magnitude as ECoG signals (Schalk and Leuthardt, 2011; Wang et al., 2017), which concurs with the fact that $\hat{n}$ is a parameter that accounts for neural activity and background noise. For a comprehensive list of $\hat{k}$ and $\hat{n}$ values across all dipoles and all subjects, the reader is referred to the Supplementary material.

**Table 3:** Median and median absolute deviation (MAD) values for dipole model parameters and R-squared values across subjects. Median and MAD are calculated across all stimulation channels in each representative grid.

**Regression**	**Summary**	**Subject 1**	**Subject 2**	**Subject 3**	**Subject 4**
**parameter**	**statistic**				
$\hat{k}\left(\Omega \text{mm}\right)$	Median	3.9	3.3	2.1	3.3
	MAD	1.2	0.3	0.3	0.9
$\hat{n}\left(mV\right)$	Median	0.070	−0.078	−0.012	0.045
	MAD	0.074	0.080	0.061	0.129
*R* ^2^	Median	0.88	0.81	0.78	0.80
	MAD	0.06	0.10	0.04	0.10

Table 3 also shows that the median values of R-squared ranged across subjects from 0.78 to 0.88. Over all subjects, R-squared values ranged from 0.33 to 0.99, with a median of 0.80 and a median absolute deviation of 0.08 (see Supplementary material for full table). This suggests that the dipole model is a good approximation for the propagation of stimulation artifacts in ECoG. R-squared values falling in the lowest 15^*th*^ percentile (*R*^2^ < 0.57) occurred due to violations of the dipole model assumptions. These include “island-like” saturation regions located away from the stimulation site, asymmetrical elongation of the saturation region along the edge of the grid, and abnormally strong artifacts appearing on electrodes adjacent to the stimulation channel.

## 4. Discussion

### 4.1. Time domain characteristics

Time-domain analysis revealed the ratcheting effect occurring in all subjects, most prominently at higher stimulation amplitudes (Figure 2). This is unsurprising given that strong stimulation amplitudes are more likely to trigger irreversible Faradaic reactions at the electrode-tissue interface (Merrill et al., 2005). These reactions generate electrochemical products during the cathodic phase that diffuse away, preventing those products from being reverted during the anodic phase. Therefore, despite the charge-balancing of the biphasic pulses, this unrecoverable loss of charges creates a residual potential. Since stimulation pulses arrive every 20 ms, which is much faster than the tissue discharge time constant (a few seconds, see Figure 2), these residual potentials accumulate over the course of a stimulation epoch to generate significant DC voltage shifts. The presence of ratcheting in a clinical, FDA-approved stimulator suggests that BD-BCI systems need to be carefully designed with superior charge-balancing mechanisms (Sohn et al., 2020; Pu et al., 2021).

The electrodes most severely affected by artifacts exhibited phase-locking of pulse responses. This is in agreement with the volume conduction assumption expressed by Equation (3). It also supports the possibility that ECoG electrodes impose consistent phase shifts whose differences fall below the signal sampling resolution of 512 Hz. To illustrate this, the model of electrode-electrolyte interface can be approximated by a parallel circuit consisting of a double-layer resistance and capacitance, serially connected to an electrolyte resistance (Merrill et al., 2005; Webster, 2009). For a typical double-layer capacitance of 10-20 μF/cm^2^ (Merrill et al., 2005) and typical resistance values in the 100–200 Ω range (Geddes et al., 1969), we estimate the ECoG electrodes' phase shifts to be in the microsecond range over the ECoG frequency band (0–200 Hz). Perturbing these parameters by a factor of 10 did not significantly change these estimates, with the largest phase shifts reaching 0.3 ms. Thus, we conclude that the differences in phase shifts imposed by individual ECoG electrodes likely fall below the sampling resolution of 1.95 ms (1/512 Hz), resulting in the appearance of phase-locked pulse responses.

### 4.2. Frequency domain characteristics

The power distribution of stimulation artifacts resembled the theoretical power spectrum of a biphasic pulse train (Figure 4). These similarities suggest that the propagation of artifacts from the stimulation channel to the recording electrodes can be approximated by a linear system. The system identification of such a model would require the simultaneous recording of stimulation pulse trains and ECoG responses. Strong stimulation artifacts at the fundamental frequency and its super-harmonics interfere with ECoG frequencies that underlie motor behavior (Wang et al., 2017; McCrimmon et al., 2018), most notably those in the γ band. Conversely, the band below the fundamental frequency exhibited little or no artifacts. This suggests that increasing the stimulation frequency above 160 Hz [the upper limit of ECoG γ band (Wang et al., 2013b)] could spare the γ band from excessive artifacts in ECoG-based BD-BCI systems. Recent experiments have demonstrated that reliable perception can be elicited in humans with stimulation frequencies as high as 500 Hz (Hiremath et al., 2017), so high-frequency stimulation might be a viable artifact mitigation strategy for BD-BCIs. However, such a high stimulation frequency would significantly increase the power consumption. This trade-off must especially be considered for fully implantable BD-BCI, where preserving the battery life may be of critical importance.

### 4.3. Spatial domain characteristics

The worst-case distance analysis gives a metric for evaluating the saturation risk of stimulation at various current amplitudes. As can be seen from Figures 5–8, at the higher stimulation amplitudes, the saturation region extends to, and possibly beyond, the edges of the ECoG grids. However, studies on artificial somatosensation (Hiremath et al., 2017; Lee et al., 2018) demonstrated that current amplitudes below 4 mA (and often times as low as 1 mA), delivered by subdurally implanted ECoG grids, were sufficient for eliciting somatosensory perception in human subjects. Saturation is thus only a concern when stimulation and recording electrodes are immediately adjacent (within 20 mm) and when the recording device has a low saturation tolerance. Optimization of other parameters such as the pulse train frequency and pulse width also permits lower-amplitude stimulation to elicit similar sensations (Hiremath et al., 2017).

The current transmission path in cortical electrostimulation depends on a number of factors. Given the complexity of the problem, we adopted a path-agnostic approach by lumping these factors into a single parameter *k* (see Equation 4). Since the units of *k* are Ωm, we can interpret this parameter as the specific resistance of the path. Furthermore, these paths, as well as the electrode properties (Webster, 2009), may depend non-linearly on the current passing through. However, our approach assumes a single regression model across a range of current amplitudes. Despite these simplifying assumptions, we still achieved a median goodness-of-fit of 0.80 across all subjects and across a wide variety of stimulation scenarios. The departure from this behavior mostly happens for stimulation channels placed on the corners of grids. In these cases, the majority of artifacts lie outside of the grid and cannot be adequately measured. Other examples of non-dipole behavior, such as the formation of “islands” or extensions of the saturation region, are potentially the result of conduction of stimulation current along neural fibers, pockets of cerebrospinal fluid, or neural vasculature. Even in many of these cases, the R-squared value is still around 0.65. Taken together, these results confirm that the spatial variations of stimulation artifacts can be explained with a simple dipole-like model. Dipole models have long been used to describe the propagation of neural signals through neural tissues (Wood, 1982; Scherg, 1990; Boon et al., 1997; Sutherling et al., 2001; Nunez and Srinivasan, 2006). However, to our knowledge there have been no other works analyzing the dipole model's applicability to ECoG stimulation artifacts. These models can be used to predict the spatial extent of artifacts and ultimately the size of the saturation region.

### 4.4. Limitations

The main limitation of our work is that the results were derived from artifacts generated and recorded by a clinical ECoG stimulation/acquisition system. To make these results generalizable to BD-BCI systems, we imposed a ±1,100 μV saturation limit based on the specifications of an implantable BD-BCI prototype (Rouse et al., 2011). Furthermore, our observations may be specific to the type of ECoG grids that were being used by the clinic. However, our analysis still encompasses grids from different manufacturers (AdTech and Integra), different grid sizes (standard and high-density), a variety implantation sites, and multiple subjects. Note that ECoG grids with similar electrode materials (platinum), size and pitch have been successfully used for motor BCI applications (Wang et al., 2013b, 2019) as well as artificial sensation studies (Hiremath et al., 2017; Lee et al., 2018). The stimulation parameters used for this study also overlap with those used to elicit artificial sensations (Lee et al., 2018) in the same ECoG grids. Therefore, despite the limitations, the results in this study are likely still applicable to BD-BCI systems.

## 5. Conclusion

This article provides a comprehensive temporal, spectral and spatial analysis of cortical electrostimulation artifacts recorded subdurally by a grid of ECoG electrodes. We have also demonstrated that the spatial distribution of stimulation artifacts can be explained by a simple dipole model. These findings can help improve existing artifact suppression techniques, inspire the development of novel artifact mitigation methods, and aid in the development of novel cortical stimulation protocols. Additionally, they may be useful for studies examining cortical functional tractography (Trebaul et al., 2018), cortico-cortical evoked potentials for clinical applications (Russo et al., 2021), and source localization for non-invasive functional neuroimaging (Mikulan et al., 2020). In general, the results in this work deepen our understanding of cortical electrostimulation and could provide critical design specifications for future BD-BCI systems.

## Data availability statement

The raw data supporting the conclusions of this article will be made available by the authors, without undue reservation.

## Ethics statement

The studies involving human participants were reviewed and approved by the Institutional Review Board of University of California, Irvine and Rancho Los Amigos National Rehabilitation Center. The patients/participants provided their written informed consent to participate in this study.

## Author contributions

JL collected the data, performed the analysis, and wrote the manuscript. PW aided in data collection and analysis. SS, HG, and MA oversaw subject-related activities and helped to perform stimulation procedures. CL performed the surgical implantations and oversaw subject-related activities. AD oversaw subject-related activities and provided feedback for the manuscript. PH provided feedback on the manuscript. ZN performed the data analysis and co-wrote the manuscript. All authors contributed to the article and approved the submitted version.

## Funding

This work was funded by the National Science Foundation (Award Nos. 1446908 and 1646275).

## Conflict of interest

The authors declare that the research was conducted in the absence of any commercial or financial relationships that could be construed as a potential conflict of interest.

## Publisher's note

All claims expressed in this article are solely those of the authors and do not necessarily represent those of their affiliated organizations, or those of the publisher, the editors and the reviewers. Any product that may be evaluated in this article, or claim that may be made by its manufacturer, is not guaranteed or endorsed by the publisher.
