# Supplementary material for: Artifact propagation in subdural cortical electrostimulation: Characterization and modeling
Source: Front Neurosci. 2022 Oct 12;16:1021097. doi: 10.3389/fnins.2022.1021097 (PMC9596776; doi:10.3389/fnins.2022.1021097)
Supplement: Supplementary file 1 [file Data_Sheet_1.PDF]

frontiers

Supplementary Material

Table S1: WCD for stimulation channels in representative grids across all subjects

Stim. Channel	WCD (mm)										
Current Amp (mA):	2	3	4	5	6	7	8	9	10	11	12
Subject 1											
LPG 4-5	12.23		12.39		12.42		12.44		12.13		
LPG 3-4	16.20		16.32								
LPG 5-10	15.10		15.19		15.26						
LPG 9 -10	12.06		12.17		12.23		12.24				
LPG 8-9	16.19		16.21		16.79		17.33				
LPG 7-8	16.05		16.12		16.13		15.49				
LPG 6-7	15.40										
LPG 1-2	15.07		18.07								
LPG 2-3	15.87										
LPG 11-12	14.83		38.01		38.34						
LPG 12-13	16.09		16.09		28.77						
LPG 13-14	16.72		17.32		17.93		25.53				
Subject 2											
LTG 1-2	14.71		15.13		15.49		17.69		18.96		
LTG 3-4	15.82		16.20		16.44		18.31		19.52		
LTG 5-10	14.35		14.63		14.82		14.92		15.01		16.95
LTG 6-7	15.13		15.57		36.45		36.17		36.15		
LTG 8-9	16.03		16.43		17.77		19.24		19.26		20.27
LTG 14-15	12.43		12.72		18.17		23.17		32.91		33.72
Subject 3											
LIHG 3-11		4.43	4.49	4.68							
LIHG 5-13		4.64	4.68								
LIHG 6-14		4.60	4.89	5.02							
LIHG 17-25		4.88		5.80		7.05		8.11	8.49		9.12
LIHG 19-27		5.11		6.48			8.07		8.71		
LIHG 20-28		4.73	4.78	5.47		6.93					
LIHG 21-29		4.65		5.01		6.10		7.34			
LIHG 22-30		4.66		7.80							
LIHG 23-30		6.30		7.20		7.79					
LIHG 7-15		5.26		5.55		6.47					
LIHG 15-23		5.38		5.51		6.24		5.10	6.95		
Subject 4											
RCG 7-8	5.40		6.90		8.12		9.60				
RCG 5-6	13.72		13.73		13.78		14.22				
RCG 3-4	6.41		10.13								

RCG 2-3	6.76		8.86								
RCG 1-2	5.92		5.91		7.08	9.78					
RCG 15-16	6.26		8.35		9.83						
RCG 13-14	8.04		9.51		10.67		11.25				
RCG 11-12	6.79		10.02		11.84		13.08		13.70		
RCG 9-10	6.04		8.69		10.49		11.87		13.96		
RCG 23-24	6.96		9.23		11.54		12.01				
RCG 21-22	7.49		10.09		10.71		11.14	11.10			
RCG 19-20	7.40		10.03		11.42		12.43		13.49	13.82	14.05
RCG 20-22	11.51		12.41		14.09		15.66		16.23		
RCG 17-18	8.26		9.89		10.19		11.54				
RCG 31-32	6.42		6.54		8.63						
RCG 29-30	7.53		8.60		9.41		10.02				
RCG 27-28	7.50		9.33		9.87		10.33		10.34		
RCG 25-26	11.95		13.30		14.30		14.84		8.13		
RCG 17-25	9.41		15.76								
RCG 18-25	9.33		13.96		13.97						
RCG 17-26	13.61		25.81		25.89						

Table S2: Dipole Fits with Coefficient Values for representative grids across all subjects

Subject 1: LPG Summary					
Stim. Channel	Stim Range (mA)	# of Epochs	$\hat{k}$ (Ω*mm)	$\hat{n}$ (mV)	R^2
LPG4-5	2–8	5	2.2	-0.109	0.66
LPG3-4	2–4	2	3.5	-0.071	0.91
LPG5-10	2–6	3	3.3	0.045	0.5
LPG9-10	2–8	4	2.4	-0.132	0.74
LPG8-9	2–8	4	2.8	-0.075	0.89
LPG7-8	2–6	4	2.8	0.031	0.87
LPG6-7	2	1	6.4	0.026	0.95
LPG1-2	2–4	2	5.7	0.056	0.88
LPG2-3	2	1	6.2	-0.011	0.99
LPG11-12	2–6	5	4.3	0.161	0.43
LPG12-13	2–6	3	5.2	-0.013	0.88
LPG13-14	2–8	4	4.9	-0.133	0.92
Subject 2: LTG Summary					
LTG1-2	2–10	10	3.2	-0.023	0.9
LTG3-4	2–10	10	3.6	0.09	0.86
LTG5-10	2–12	12	2.8	0.049	0.76
LTG6-7	2–10	10	3.4	-0.099	0.67
LTG8-9	2–10	10	3.5	0.125	0.91
LTG14-15	2–12	12	2.8	0.407	0.68
Subject 3: LIHG Summary					
LIHG1-9	3–4	2	2.6	-0.054	0.81

LIHG3-11	3–5	3	2	0.001	0.87
LIHG5-13	3–4	2	2.4	0.035	0.75
LIHG6-14	3–5	3	2.1	0.127	0.81
LIHG17-25	3–12	6	1.5	-0.244	0.72
LIHG19-27	3–10	5	1.8	-0.19	0.77
LIHG20-28	3–7	4	2.1	-0.112	0.77
LIHG21-29	3–9	5	1.8	-0.113	0.78
LIHG22-30	3–5	4	2.3	-0.084	0.8
LIHG23-31	3–7	4	2.5	-0.179	0.82
LIHG7-15	3–7	3	1.8	0.003	0.67
LIHG15-23	3–10	6	1.1	-0.072	0.57
Subject 4: LIHG Summary					
RCG7-8	2–8	4	1.7	0.425	0.67
RCG5-6	2–8	4	4.5	-0.105	0.46
RCG3-4	2–8	4	3.5	-0.137	0.8
RCG2-3	2–4	2	4.8	-0.052	0.88
RCG1-2	2–7	4	2.3	-0.064	0.79
RCG15-16	2–6	3	2.6	0.324	0.92
RCG13-14	2–6	4	3.3	0.15	0.72
RCG11-12	2–10	4	3.5	0.059	0.74
RCG9-10	2–10	6	3.3	-0.084	0.75
RCG23-24	2–8	4	2.3	0.457	0.86
RCG21-22	2–9	5	3	0.058	0.91
RCG19-20	2–12	7	3.3	0.045	0.91
RCG20-22	2–10	5	2.9	0.127	0.89
RCG17-18	2–8	5	4.2	-0.12	0.86
RCG31-32	2–6	3	2.3	0.256	0.74
RCG29-30	2–8	4	3.5	0.129	0.95
RCG27-28	2–10	5	3.1	-0.004	0.87
RCG25-26	2–10	5	2.1	-0.257	0.33
RCG17-25	2–4	2	9.2	-0.149	0.37
RCG18-25	2–6	3	6.7	0.131	0.46
